# Restoring a Partial Maxillectomy Patient by an Implant-Supported Obturator on Two Implants: A Case Report

**Published:** 2018-05

**Authors:** Elaheh Beyabanaki, Marzieh Alikhasi

**Affiliations:** 1 Assistant Professor, Department of Prosthodontics, School of Dentistry, Shahid Beheshti University of Medical Sciences, Tehran, Iran; 2 Associate Professor, Dentistry Research Institute, Tehran University of Medical Sciences, Tehran, Iran; Department of Prosthodontics, School of Dentistry, Tehran University of Medical Sciences, Tehran, Iran

**Keywords:** Dental Implants, Maxillary Neoplasms, Implant-Supported Dental Prosthesis, Maxillofacial Prosthesis

## Abstract

This article describes the prosthetic treatment of a patient suffering from a hemimaxillary defect after surgical resection of an adenoid cystic carcinoma (ACC) in the palate. The patient had also received therapeutic irradiation. One year after radiotherapy, three implants were placed in the remaining maxillary bone without any bone augmentation. One of the implants failed during the osseointegration period. The implant replacing the failed one also failed during prosthetic procedures. The patient was unwilling to undergo another surgical episode, and the final prosthesis was completed on the two remaining implants.

## INTRODUCTION

Dental implants improve the retention, stability, and support of prostheses in edentulous patients [1]. They are especially valuable for edentulous patients who have lost a segment of either jaw due to resection of a tumor [1]. Sometimes, surgical resections should be followed by irradiation to ensure complete eradication of a malignant or recurring tumor [2]. However, there can be concerns about the quality of the bone that has received radiotherapy in terms of osseointegration [3]. Radiotherapy following tumor resection can adversely affect the bone-implant contact [3,4].

Implant-supported obturators for maxillectomy patients have some benefits over conventional obturators [5,6]. However, little data exists on the proper number, length, diameter, and distribution of implants throughout the remaining arch. This lack of clarity might be related to patient-specific conditions in terms of the defect size and available bony sites. According to Roumanas et al [7], four implants have been suggested for implant-supported obturators. Furthermore, the most suitable sites suggested for dental implant placement is the remaining premaxillary segment as well as the posterior edentulous process near the tuberosity [8]. However, the ideal implant placement might be compromised by resected sites, inadequate remaining bone, and irradiated tissues which would result in a less effective anterior-posterior distribution and cross-arch stabilization patterns [9,10]. Zygomatic implants are also a simple and cost-effective option for some maxillectomy patients [11].

There are several factors that could affect the predictability of a maxillary implant-supported overdenture including the quality and the amount of the remaining bone, the potential location of implants, esthetics, and phonetics [12,13]. These factors could also be applicable to implant-supported obturators in maxillectomy patients. It has also been reported that the survival rate of maxillary implant-supported overdentures is 96.1% [14], which is less than that of their mandibular counterparts [15,16]. This finding is likely more evident for implant-supported obturators because some of the support, retention, and stability potential is lost due to the maxillectomy procedure. The incidence of implant failure is allegedly higher for maxillary overdentures [15,16], and can be even higher when obturators are used. Generally, there are more extensive data regarding mandibular implant-supported overdentures in comparison with their maxillary counterparts [17,18]. The minimum offered implant-supported prosthesis in an edentulous mandible is a two-implant supported overdenture [17–19]. However, it has been suggested that mandibular overdentures supported with only one implant in the midline of the arch could lead to a successful result [20,21]. According to Pan et al [22], the arch anatomy such as its height is not an absolute predictor of patient satisfaction with either conventional or implant-supported dentures in the mandible. It seems that the same reasoning regarding the number of implants might be true for the maxillary arch depending on some factors such as occlusal forces (opposing arch dentition), the location of implants, and the patient’s bite force. Also, according to the literature, the lowest survival rate among implant-supported prostheses belongs to maxillary overdentures [15,16]. There are no data on the survival rate of implant-supported maxillary obturators. Nevertheless, such prostheses could be a precious treatment modality in these patients and could improve their function, esthetics, and comfort. However, considering the lower quality and quantity of bone in most of these patients due to previous radiation and bone resorption, the treatment might be less than optimal. This article describes the treatment of a maxillectomy patient by two dental implants.

## CASE REPORT

A 53-year-old man was referred to the Prosthodontics Department of Tehran University of Medical Sciences, one and a half years after surgical resection and radiotherapy of an adenoid cystic carcinoma (ACC) in the right side of the maxillary arch by a dose of 45 Grays (Gy). The patient was completely edentulous and dissatisfied with the retention and function (nasal reflux) of his existing maxillary obturator opposing a mandibular denture.

**Fig. 1:**
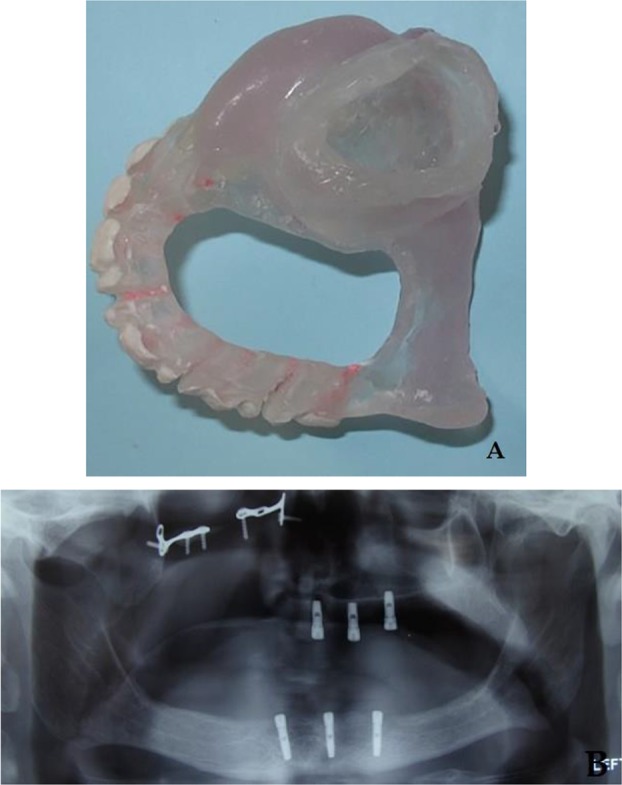
(A) Maxillary surgical template. (B) Panoramic view of the implants inserted in the upper and lower jaws

The patient requested implant-supported maxillary and mandibular prostheses. The most suitable sites for implant placement were determined with the aid of cone-beam computed tomography (CBCT), and the patient’s existing dentures were duplicated for fabricating radiographic stents. Three dental implants (Implantium®, Dentium, Seoul, South Korea) with the diameter of 3.5 mm and the length of 10 mm in the maxilla and 12 mm in the mandible were inserted in the jaws without any bone augmentation after converting radiographic templates into surgical ones (Fig. 1). The existing dentures were then relined by using a soft liner (Mollosil®, Detax Dental GmbH & Co. KG, Ettlingen, Germany) to relieve the pressure on the implants and to create a better fit with the underlying tissues during the osseointegration period. Six months later, during the second surgery, the most distal implant of the upper arch was removed due to the lack of osseointegration.

**Fig. 2:**
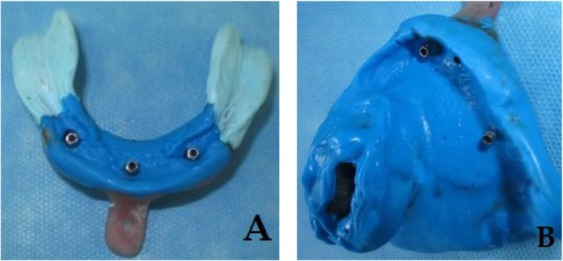
Final impressions of the (A) lower and (B) upper jaws by using the open tray technique

Two weeks later, another implant was placed instead of the failed implant but at a slightly more distal site. After another three months, the last implant was uncovered, and a healing abutment was secured. The presence of an acceptable osseointegration was confirmed clinically by torque test (Osstell^TM^, Mentor, Integration Diagnostics AB, Sävedalen, Sweden) and x-ray radiography. Two weeks later, primary impressions were made by using an irreversible hydrocolloid impression material (Alginoplast, Heraeus Kulzer GmbH & Co., Wehrheim, Germany) and prefabricated trays (Dandal, Taksan, Tehran, Iran). Final impressions were made by using splinted square impression copings with custom trays [23] and regular body polyvinyl siloxane (PVS; Panasil® monophase Medium, Kettenbach GmbH & Co. KG, Schoenberg, Germany) for the maxilla and with a combination of zinc oxide eugenol (ZnOE, Cavex, Holland and IRM, Dentsply, USA) and regular body PVS for the mandible (Fig. 2). After determining the vertical dimension of the occlusion, the space analysis of the upper and lower arches indicated the necessity of choosing individual stud attachments with limited height requirements [24]. At this time, the patient declared pain during mastication; however, there were no other clinical or radiographic signs other than pain upon percussion.

After consulting with the surgeon, the treatment was continued by considering the possible failure of the suspicious implant. To compensate for the minimal implant divergence and to create a definite path of insertion for the obturator that would accommodate both attachments and the extension of the prosthesis into the defect (bulb), 15° angled abutments (Kerator, Daekwang IDM Co., Seoul, South Korea) were selected. Straight attachments (Positioner, Implantium®, Dentium, Seoul, South Korea) were used for the mandibular overdenture. While the wax-up was being prepared (two weeks after the initial symptom), the patient declared constant pain. Upon opening the healing abutment for further examination, the implant was removed. The patient was unwilling to receive any further surgical procedure, and implant replacement was not followed due to the failure risk [25]. Therefore, the treatment was continued with the two remaining implants in the maxillary arch, and the frameworks were fabricated.

**Fig. 3:**
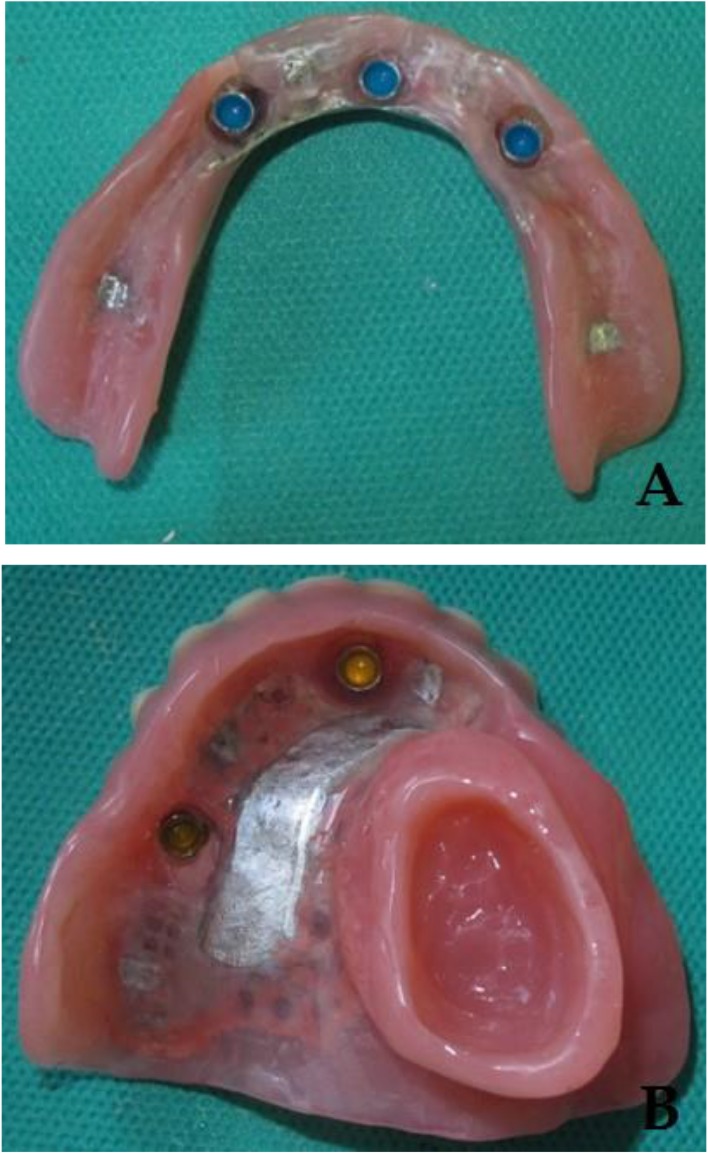
Tissue surfaces of the (A) upper and (B) lower prostheses

**Fig. 4:**
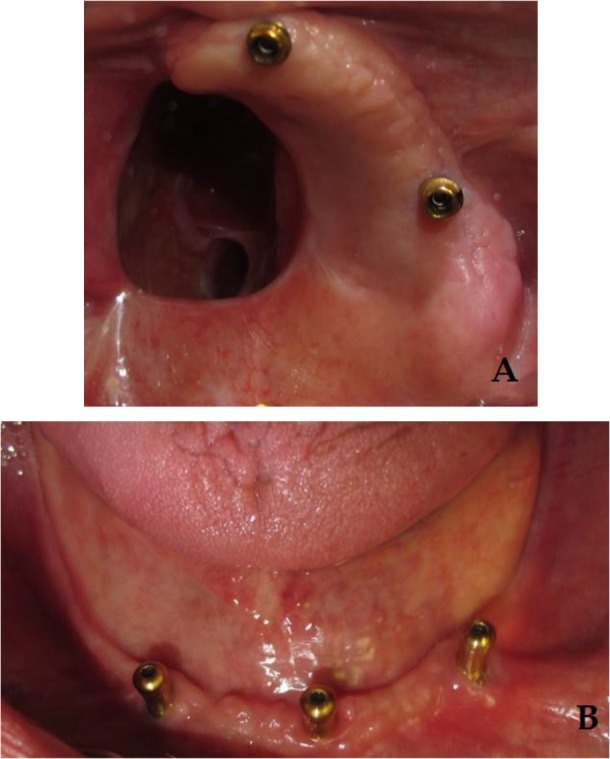
Intraoral views of the stud attachments connected to the implants in the (A) upper and (B) lower jaws

Before processing the prostheses, the next visit was managed for another try-in with the frameworks placed in record bases. After confirming all the parameters in the try-in session (the vertical dimension of the occlusion, esthetics, phonetics, and the centric relation), the prostheses were processed (Fig. 3). At the delivery visit, the abutments were secured in the mouth with 30-newton centimeter (N/cm) torque according to the manufacturer’s recommendation (Fig. 4), and the prostheses were checked and adjusted. The patient received oral hygiene instructions and a recommendation to wear the obturator at night for managing mucosal and salivary secretions [26,27]. Subsequently, a panoramic radiograph was taken as a baseline for future evaluations. Some adjustments were needed during follow-up visits. After two years, regular six-month follow-ups showed acceptable conditions of the implants and the prostheses. Long-term observations will be used to ensure the patient’s oral health and the competence of the prostheses.

## DISCUSSION

There is limited scientific evidence regarding implant-supported obturators in edentulous maxillectomy patients. However, clinical experience clearly justifies the use of dental implants for improving the support, retention, and stability of the prostheses in these patients. Depending on the patient’s situation, it is not always possible to deliver the perfect treatment plan. One of the factors that might hinder the clinician from delivering an optimal prosthetic treatment is the remaining bone. The factors affecting the implant placement in an edentulous bone after surgical resection include the quality, quantity, and position of the residual bone [9,10,15,16].

Despite using a surgical stent, the implants were not completely parallel due to the lack of optimal bone volume and the patient’s disinterest in further surgery for bone augmentation. Also, in order to achieve a definite path of placement in accordance with the defect’s undercuts, angled Kerator attachments were chosen. These attachments provide more retention in comparison with ball attachments [28].

According to the literature, there is generally no difference among different retentive mechanisms in implant-supported dentures in terms of treatment success [29]. Additionally, the index taken from the palatal side after the tooth setup showed that if a bar attachment was used for connecting two relatively far implants, its bulk would have interfered with the palatal segment of the prosthesis. Also according to this index, an attachment with minimum height was needed for the posterior implant.

The reason for considering an open hollow bulb for the obturator was to decrease the weight of the prosthesis. Also, the fabrication and adjustment of an open hollow obturator are easier and more common in comparison with a closed one [26,30, 31]. However, the only problem is the accumulation of food and nasal secretions inside the hollow part, which would not cause a problem if the walls of the bulb are made free of undercuts [26].

The patient did not have ideal conditions for an implant-supported obturator because of insufficient bone in terms of height, width, angulation, and quality. He had been edentulous for a long time before tumor surgery, and the bone was exposed to irradiation after the surgery. The patient was reluctant to receive any additional bone augmentation surgery. Therefore, to increase the bone width, a bone spreading technique was performed. However, despite this technique, one of the implants failed during osseointegration. This could be because of a lack of proper bone quality and quantity. Furthermore, irradiation could have added to the inherently lower quality of the maxillary bone versus the mandibular counterpart. Therefore, it might be inevitable to deviate from the standard treatment principals with regard to the number of implants placed in edentulous jaws. Examples of such deviations suggest patient satisfaction with placing one implant in the midline of the mandible [20,21] and one implants in the maxillary arch for a palate-free overdenture [27].

Although it seems that using only two implants for an implant-supported obturator is far from the scientific guidelines, it may fit the numerous suboptimal situations that clinicians might face during the treatment of maxillofacial patients. Since these patients benefit from dental implants, depriving them of such treatments due to the lack of optimal conditions for placing the presumed number of implants would not be necessarily justified. Therefore, further research is warranted to definitively confirm this approach.

## COUCLUSION

This clinical report describes the implant-supported prosthetic treatment of a maxillofacial patient with three implants in the maxilla and subsequently two implants due to the failure of one of the implants during the prosthetic phase. The final prosthesis provided acceptable retention, stability, and patient satisfaction. However, the literature lacks definite evidence to disprove such a treatment considering the suboptimal conditions in maxillofacial patients for providing the optimal number of implants. Therefore, standardized studies are needed to confirm this approach.
